# The dominance–diversity dilemma in animal conservation biology

**DOI:** 10.1371/journal.pone.0283439

**Published:** 2023-03-27

**Authors:** Charles A. Martin, Christopher J. Watson, Arthur de Grandpré, Louis Desrochers, Lucas Deschamps, Matteo Giacomazzo, Audréanne Loiselle, Cindy Paquette, Marc Pépino, Vincent Rainville, Guillaume Rheault, Raphaël Proulx

**Affiliations:** 1 Université du Québec à Trois-Rivières, Trois-Rivières, Québec, Canada; 2 Centre for Research on Watershed–Aquatic Ecosystem Interactions, University of Québec at Trois-Rivières, Trois-Rivières, Québec, Canada; 3 Université de Montréal, Montréal, Québec, Canada; 4 Institut de Recherche en Biologie Végétale, Montréal, Québec, Canada; 5 Université du Québec à Montréal, Montréal, Québec, Canada; 6 Ministère des Forêts, de la Faune et des Parcs, Direction de la Gestion de la Faune Mauricie–Centre-du-Québec, Trois-Rivières, Québec, Canada; 7 Parcs Canada, Shawinigan, Québec, Canada; Southeastern Louisiana University, UNITED STATES

## Abstract

The alteration of environmental conditions has two major outcomes on the demographics of living organisms: population decline of the common species and extinction of the rarest ones. Halting the decline of abundant species as well as the erosion of biodiversity require solutions that may be mismatched, despite being rooted in similar causes. In this study, we demonstrate how rank abundance distribution (RAD) models are mathematical representations of a dominance-diversity dilemma. Across 4,375 animal communities from a range of taxonomic groups, we found that a reversed RAD model correctly predicts species richness, based solely on the relative dominance of the most abundant species in a community and the total number of individuals. Overall, predictions from this RAD model explained 69% of the variance in species richness, compared to 20% explained by simply regressing species richness on the relative dominance of the most abundant species. Using the reversed RAD model, we illustrate how species richness is co-limited by the total abundance of a community and the relative dominance of the most common species. Our results highlight an intrinsic trade-off between species richness and dominance that is present in the structure of RAD models and real-world animal community data. This dominance-diversity dilemma suggests that withdrawing individuals from abundant populations might contribute to the conservation of species richness. However, we posit that the positive effect of harvesting on biodiversity is often offset by exploitation practices with negative collateral consequences, such as habitat destruction or species bycatches.

## 1. Introduction

Our planet is undergoing rapid changes, including global climate modification, large scale habitat conversion, overexploitation of animal populations and unprecedented levels of pollution [1]. All these changes have two major outcomes on the demographics of living organisms: the decline of common species and the extinction of rarest ones. For example, the Living Planet Index (LPI) tracks over 16,700 animal populations across the world, many of which are exploited populations. The 2018 LPI report emphasizes the decline of demographic indices by 60% on average across species over the last 50 years ([2], but see [3] for a more nuanced analysis). Similarly, Rosenberg et al. [4] estimated that since the 1970s, the North American avifauna has lost over 3 billion birds, which corresponds to a decrease of 29% of its overall abundance, predominantly in common species. At the other end of the dominance-diversity spectrum, the IUCN Red List has been developed to monitor global trends in species extinctions, while acknowledging the increasing efforts deployed in species assessment [5]. Global analysis of this list shows that, in all taxonomic groups, the risk of rare species extinction is constantly increasing since the 1980s [e.g. 6, 7]. The rate of biodiversity erosion is so high that many scientists are now calling the current situation “the sixth mass extinction” [8].

Although both population decline and species extinction are ultimately rooted in similar causes, practitioners often face trade-offs when choosing where to prioritize resources. For instance, a large-scale study quantified the opportunity costs of conserving rare species of migratory fishes in the context of removing weirs and increasing habitat connectivity across thousands of tributaries [9]. Prioritizing projects to maximize benefits for the rarest species led to the poorest average habitat gains for other species, especially common ones [9]. Another study using abundance data of 144 bird species reported that European bird populations declined by 50% between 1980 and 2009, while the abundance of less-dominant species increased over the same period, presumably as the result of targeted conservation programs [10]. These counterintuitive results suggest the existence of a dominance-diversity trade-off in some situations, where the conservation of rare species parallels the decline of common ones and *vice versa*.

The dominance-diversity trade-off is also pervasive in the structure of ecological communities. Species assemblages share a common structure characterized by the presence of a few dominant and many rare species [11]. One simple way of representing this structure is to rank species according to their relative abundance in the community (rank abundance distributions; RADs). Over the last decades, ecologists have proposed several abundance distribution models [see 11], which range from resource apportionment models (e.g., dominance pre-emption and MacArthur random fraction) to purely semi-parametric distributions (e.g., log- and geometric-series, lognormal and Zipf-Mandelbrot). Many studies have attempted to identify which of these models best captures the structure of ecological communities, often with mitigated success [12–14]. Although abundance distribution models are tailored to reflect different ecological processes, their shared mathematical structure suggests the existence of a conservation compromise, where managing for abundant species and species richness cannot be achieved at once.

The objective of this study is to explicitly demonstrate how RAD models are mathematical representations of the dominance-diversity dilemma. Specifically, we evaluate whether RAD models can successfully predict animal species richness from the relative dominance of the most abundant species in thousands of communities across a range of taxonomic groups. We then discuss the implications of the dominance-diversity dilemma for stock management and biodiversity conservation.

## 2. Methods

### 2.1 Inversion of RAD models

RAD models can be fitted to observed species abundances, with the underlying parameter values varying freely from one community to the other. In a few cases, the probability of observing a species depends only on the total number of individuals and species richness, such as in random apportionment [15] and geometric-series (GS) models. In principle, these simpler models can be inverted to predict species richness from a dominance index only. More sophisticated RAD models are less easily inverted as they require the estimation of free parameters, which are obtained by fitting the observed data, leading to circular reasoning. For this paper, and without loss of generality, our efforts are focussed on the GS and log-series models.

The GS model can be described as an iterative process, where each new species arriving in a community takes a given fraction (*k*) of the remaining resources. The first species thus takes a fraction *k* of the resources, the second one *k* (1-*k*), and so on. Implicit in the GS model is the notion that resource preemption results in a directly proportional abundance for each species in a community. A species taking 40% of the resources will represent 40% of the individuals, etc. He & Tang [16] showed that the community parameter *k* can be precisely estimated by simply knowing species richness (*SR*) and the abundances of the least (*Nmin*) and most (*Nmax*) abundant species as follows:

$$k={1-(\frac{Nmin}{Nmax})}^{\frac{1}{SR-1}}.$$ (1)

This equation can be transformed to isolate *SR*, *giving*:

$$SR=\frac{ln(\frac{Nmin}{Nmax})}{ln(1-k)}+1.$$

Doing so, *SR* goes from an observed quantity to an estimated quantity in the model, but *k* is still an estimated parameter. We therefore approximate *k* by *MaxRel*, the relative abundance of the most abundant species (i.e., *MaxRel* = *Nmax* / *Ntot*, where *Ntot* is the total number of individuals), giving the following model:

$$SR=\frac{ln(\frac{Nmin}{MaxRel\cdot Ntot})}{ln(1-MaxRel)}+1.$$

Finally, as *Nmin* is more an artefact of the sampling method than a community property *per se* and given the fact that it is equal to 1 in 83% of the studied communities, we also replace *Nmin* by 1, giving the complete reversed GS model as:

$$SR=\left[\frac{ln(\frac{1}{MaxRel\cdot Ntot})}{ln(1-MaxRel)}\right]+1.$$ (2)

An alternative version of the inverted model, without the *Nmin* = 1 simplification, is presented in S1 Appendix with results that are almost indistinguishable from those produced by Eq 2. Using Eq 2, it is possible to predict the *SR* of a community by knowing only its relative dominance value (*MaxRel*), along with the total number of individuals in the sample (*Ntot*).

The GS is not the first abundance distribution model inverted to predict *SR*. Fisher’s log-series (LS, [17]) is a statistical distribution that fits the number of species (x-axis) in a community with a particular number of individuals (y-axis). The LS model was evaluated on thousands of ecological communities [18, 19] and is represented by the following equation:

$$\Phi (n)=\alpha (x^{n}/n)$$ (3)

where Φ(*n*) represents how many species of *n* individuals are expected in a sample, *α* is Fisher’s diversity parameter and *x* is a constant close to 1. Fisher proceeds to show that the species richness of a community is approximated by the following equation:

$$SR=\alpha \;ln(1+N_{tot}/\alpha )$$ (4)

where *Ntot* is the total number of individuals in the sample and *α* is the number of species observed only once. It was later demonstrated that Eq 4 produces better predictions of *SR* than non-parametric models [20] and realistic estimates in species rich communities [21, 22]. However, predicting *SR* from Eq 4 is somewhat circular because one needs the number of singleton species, which is obtained by identifying all species in the community, i.e. *SR* is already known. Nevertheless, the LS model represents a good benchmark to assess how well the inverted GS model performs. Therefore, we assessed the fit of both models for comparison purposes.

### 2.2 Dataset preparation

The BioTIME dataset [23] contains a large collection of ecological communities across the globe and aims at evaluating temporal trends of biodiversity in ecological assemblages. To explore the idea of predicting the species richness of a community through the relative dominance of its most common species, we downloaded the full BioTIME dataset on December 12 2019 and filtered it to keep only observations from animal communities (see Table 1 for a per taxa breakdown). We excluded plants because *Nmax* and *Ntot* are ill-defined when biomass or cover are used as abundance measures. Similarly, we eliminated communities in which *Nmin* and *Nmax* were equal (i.e. maximum evenness) because the GS model is not properly defined for these limit cases.

**Table 1:** Per-taxa breakdown of community data and bias assessment metrics.

Taxa	MAE*	MBE*	n	*SR*
Benthos	1.53 (1.47–1.61)	1.34 (1.26–1.42)	177	30.8
Birds	1.37 (1.35–1.40)	0.82 (0.80–0.85)	546	15.9
Fish	1.55 (1.52–1.58)	1.33 (1.30–1.37)	1,168	22.6
Mammals	1.42 (1.39–1.45)	0.77 (0.75–0.79)	626	4.2
Marine invertebrates	1.45 (1.43–1.47)	1.04 (1.02–1.07)	1,613	12.7
Terrestrial invertebrates	1.22 (1.20–1.25)	0.98 (0.95–1.02)	231	6.6
Other	1.15 (1.08–1.25)	0.97 (0.87–1.06)	14	3.8

Corrected mean average error (MAE*), corrected mean bias error (MBE*) with 95% non-parametric bootstrap confidence intervals (from 1000 resampled datasets), number of communities (n) and average species richness (SR) by taxa. Errors are back-transformed means of log-log residuals of the corrected geometric-series model. Other category includes amphibians (n = 1), freshwater invertebrates (n = 2) and reptiles (n = 11).

RAD models assume that species interact and share a common set of resources. To prevent the inclusion of communities that are mainly structured by environmental heterogeneity rather than competition for resources, we restricted our analyses to studies conducted with a spatial grain less than 1 km^2^. We acknowledge that this cut-off is large and that processes other than resource partitioning could still cause some of the observed variation in species richness. Nevertheless, we assume that the authors of the original studies identified the proper spatial grain for their target organisms. Finally, we used the most recent data point of each survey, resulting in a final dataset of 4,375 independent local animal communities. We hereby adopted an inclusive definition of a “local community”, which is a collection of individuals from different species that share (i.e. are sampled in) the same area, over the same period, using a common sampling protocol. For each community, we calculated the relative dominance of the most common species and used it in combination with total abundance to predict *SR* as per Eq 2.

### 2.3 Error magnitude and direction assessment

We calculated the prediction error around *SR* estimates from the inverted GS model as a log-log residual, i.e, *r = log (SR*_*observed*_*)–log (SR*_*gs*_*)*, where *SR*_*observed*_ is the species richness of each community and *SR*_*gs*_ is the species richness estimated through the inverted GS model.

To assess the magnitude of the prediction error, we calculated the mean absolute error (MAE), and then back-transformed it so that it could be interpreted as an error ratio:

$$MAE=e^{\sum_{i=1}^{n}\frac{\left|r\right|}{n}}$$

where *r* is the log residual species richness of each community. A MAE of 1 means perfect prediction while, for example, a MAE of 1.3 means, on average, 30% error.

We calculated the direction of errors (i.e. systematic bias) as the mean bias error (MBE), and also back-transformed it to ease the interpretation:

$$MBE=e^{\sum_{i=1}^{n}\frac{r}{n}}$$

The further MBE is from 1, the more biased are the residuals to a particular side. A value of MBE larger than 1 means positive residuals, thus an underestimation of *SR*. Conversely, MBE values smaller than 1 means negative residuals, thus a systematic overestimation.

### 2.4 Bias correction

The GS model is known to be a steeply decreasing RAD that underestimates the *SR* of species-rich communities. To estimate an empirical correction factor (*cf*) that could reduce this bias, we fitted the following least-square model:

$$log(SR_{observed})=cf\times log(SR_{gs})$$

This model was adjusted using the *lm* function in R [v. 4.1.1; 24]. Residuals were visually assessed for normality and homoscedasticity.

## 3. Results

**Fig 1:**
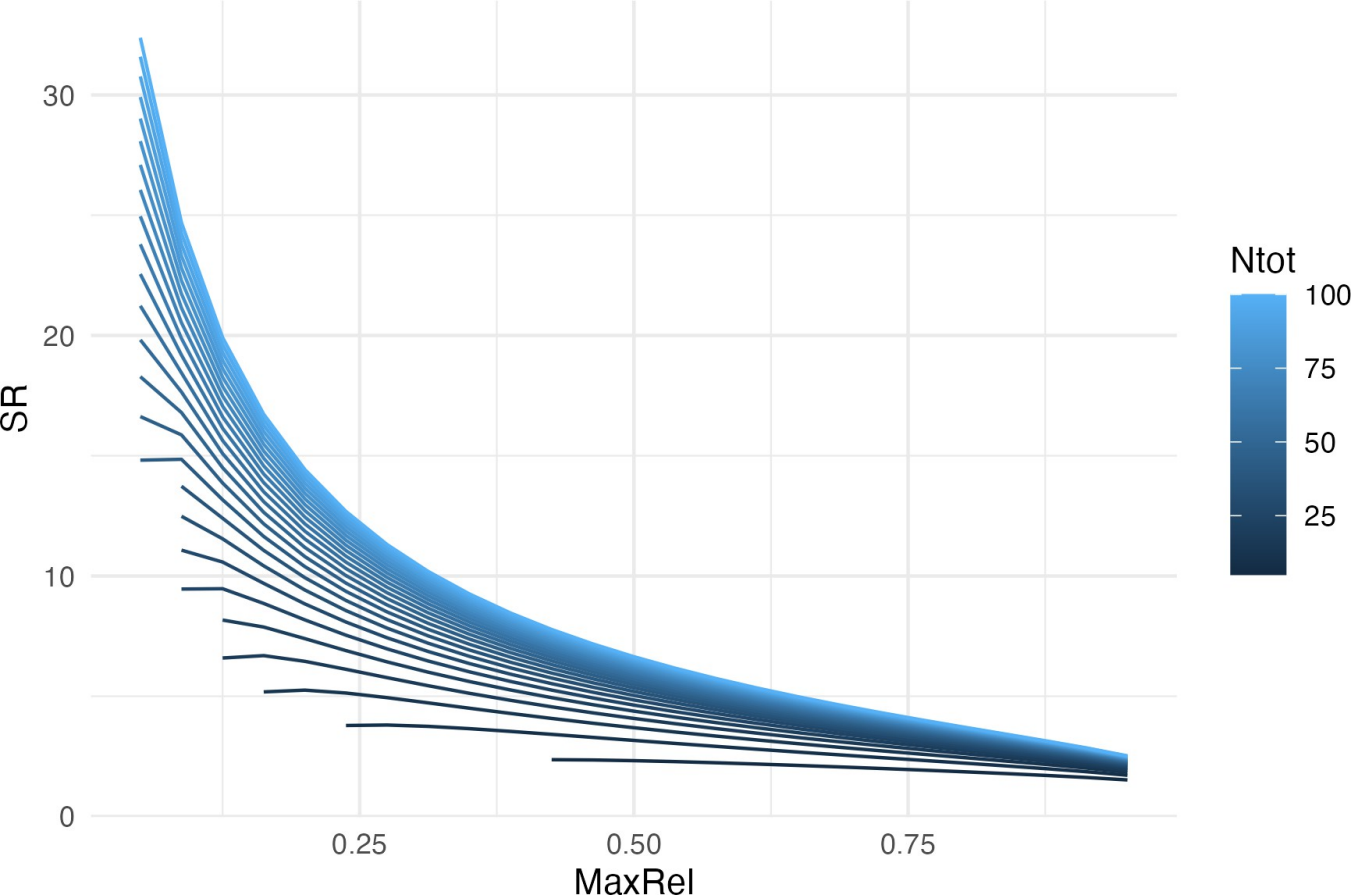
Theoretical relationship between species richness and dominance in a community. *SR* is the species richness value, *MaxRel* represents the relative abundance of the most common species and *Ntot* represents the total number of individuals in the community. Simulations were conducted using Eq 2 and a grid of parameter values where *MaxRel* ranged from 0.05 to 0.95 and *Ntot* ranged from 5 to 100 individuals. Communities where dominant species had less than 2 individuals were removed because these communities could not be dominated *per se*.

The general dominance-diversity dilemma is illustrated by simulating *SR* from Eq 2 using different values of *MaxRel* and *Ntot* (Fig 1). When the number of sampled individuals (*Ntot*) is high, *SR* decreases with increasing *MaxRel*. At lower values of *Ntot*, the relationship between *SR* and *MaxRel* becomes increasingly flat, indicating that both the community size and the sampling effort affects the prediction of *SR* (Fig 1).

**Fig 2:**
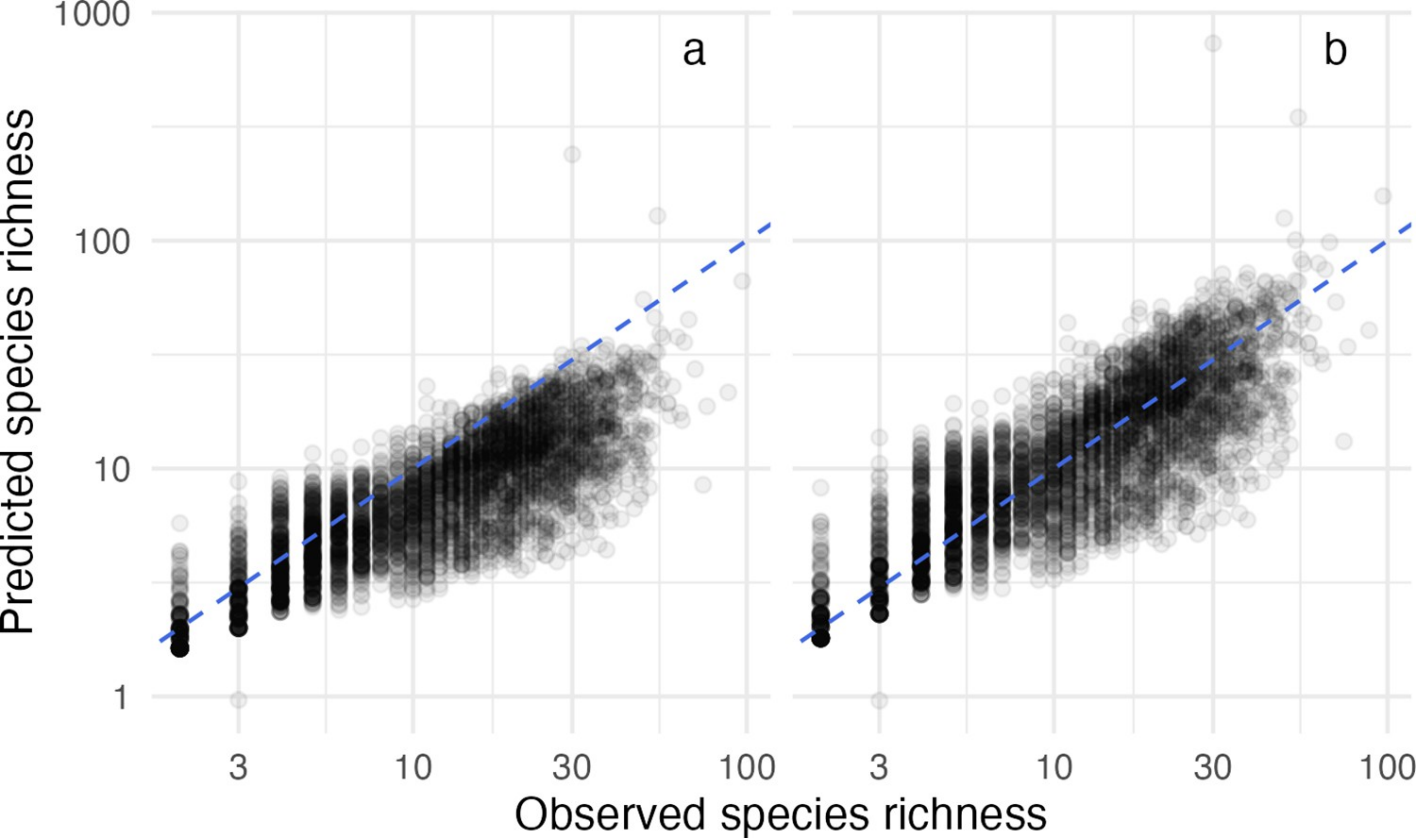
Relationship between the observed species richness and model predictions in 4,375 animal communities. With the raw predictions from a geometric-series model (a) and after applying an empirical correction factor (b). Dashed line is the 1:1 relationship.

We predicted the *SR* of 4,375 local communities from around the world using only the relative dominance (*MaxRel*) and the total number of sampled individuals (*Ntot*). The relative dominance across all datasets and communities ranged from 0.061 to 0.995, with the median at 0.448. The inverted GS model successfully predicted *SR* in actual communities, as assessed by the squared correlation between log-predicted and log-observed *SR* (pseudo-r^2^ = 0.69; Fig 2A). By comparison, the pseudo-r^2^ of the log-log empirical relationship between *MaxRel* and observed *SR* was much lower at 0.20. Although our predictive approach presented a reasonable fit to the observed data, the global error magnitude is nonetheless relatively high, with a MAE of 1.66. Using a comparable dataset (i.e., with only communities containing at least one singleton species, n = 3,665), the pseudo-r^2^ of the LS model is 0.82. The pseudo-r^2^ of the log-log empirical relationship between the number of singleton species in a community (Fisher’s *α*) and observed *SR* was also much lower at 0.60. Inversion of GS or LS models always produced better approximations of *SR*.

As expected from its steeply declining nature, the GS model fit was systematically biased (Fig 2A). On average, the inverted RAD model underestimated *SR* (MBE = 1.56), especially for species-rich communities. We found that multiplying *log (SR)* by a correction factor (*cf*) of 1.20 (+/- 0.004 SE) both reduced error magnitude (MAE* = 1.45) and overall cancelled the systematic bias (MBE* = 1.04) (Fig 2B, see Table 1 for a per taxa breakdown). This empirical *cf* was obtained at no extra cost of input variables and can be factored in Eq 2 directly. However, systematic bias > 30% could still be detected for fish and benthos, even after applying the *cf*.

## 4. Discussion

RAD models are frequently used to describe the structure of ecological communities, but their mathematical underpinnings hide additional ecological insights. We showed that the inversion of the simple GS model can be used to approximate *SR* with a minimum of information on the size of the community and the relative abundance of the dominant species. Consequently, our results suggest the existence of an intrinsic trade-off between dominance and diversity (i.e. the long tail of locally rare species in RADs), irrespective of taxa or sampling protocols. Hints about the existence of that trade-off have been reported before, either directly for fish and lizard communities [25, 26], or indirectly for foraminifera [27]. However, in all the above studies, dominance was treated as a reciprocal measure of biodiversity, and not as a driving force, such as implied by the structure of inverted RAD models.

Moreover, inversion of the GS model better predicts *SR* than simply regressing it against dominance. Indeed, the pseudo-r^2^ of the model inversion approach is 69% in comparison to 20% for the empirical regression between *SR* and dominance. However, with 45% error on *SR* estimates, inversion of the GS model does not provide the level of precision needed for surveying individual animal communities. For example, the *SR* of a 20-species community predicted by the inverted GS model (including the correction factor) could fall anywhere between 12 and 23 species (first and third quartiles for 113 communities of exactly 20 species; Fig 2B). Furthermore, considerable bias asymmetry persisted for some taxonomic groups like small mammals and some aquatic organisms, which would warrant a more in-depth analysis. Factors such as the type of sampling protocol, the regional species richness, or animal behaviour could strongly influence the predictive ability of the inverted GS model.

Approximation of *SR* through the LS model slightly outperforms the inverted and corrected GS model. This result was expected since the LS model requires more data to parameterize, as all the singleton species in a sample must be identified to predict *SR*. By comparison, the parameters of the GS model only require the relative abundance of the dominant species. From a conceptual standpoint, the GS model captures the iterative process of resource pre-emption by competing species, whereas the LS model is often regarded as a purely statistical representation of the community (but see [18] for a process-based interpretation of the LS model).

Inversion of the GS model suggests that factors affecting the size of the community, irrespective of dominance, also have an influence on *SR*. For a given dominance value, increasing the abundance of the entire community (e.g., through habitat restoration) should increase *SR*, whereas decreasing it should decrease *SR* (Fig 1). Our approach therefore decouples the effect of relative dominance among species from the effect of total abundance. The dominance-diversity trade-off reveals itself only by altering relative dominance *per se*. Consequently, one can predict that selective harvesting of dominant species should increase *SR*, whereas indiscriminate harvesting of individuals across the entire community should decrease it.

The dominance-diversity trade-off is in line with current knowledge of invasive species management. Release of exploitative pressure on invasive species upon their introduction in a new location may induce dominance (aka enemy-release hypothesis, [28]) and cascade negatively on the *SR* of host communities [29]. The inverted GS model supports this mechanism and provides testable predictions of species losses following the introduction of a dominant invader. Conversely, the approach could also be used to compare different management scenarios, in order to prioritize interventions in locations where eliminating or controlling an invasive species might have the largest positive effects on biodiversity.

Implications of the dominance-diversity trade-off for the exploitation of natural populations are less intuitive. For instance, halting the harvest of an abundant fish stock may help restore densities and increase the long-term viability of that population. Although one might expect that the whole community should benefit from such no-catch policies, the inversion of the GS model suggests that, overall, one might see a decrease in *SR* in those areas due to increased dominance. This could explain why some no-catch marine reserves reported negative effects on *SR* in comparison to nearby exploited communities, despite good protection levels and ongoing habitat alterations outside reserves ([30], negative effects are reported for 11 out of 39 studies in S1 Table therein).

Conservationists may be tempted to propose harvesting the most abundant species in a community to support *SR* in exploited ecosystems. And indeed, exploitation or experimental removal of abundant species have been shown to increase the diversity of local communities, especially if leftover resources become available to rare species [31–33]. However, in a hyper-connected world, there is a real possibility that the resources freed by such a harvest would allow another opportunistic species to enter the community and reinforce, or even worsen, the dominance pattern. Also, it is important to emphasize that most exploitation methods, especially commercial ones, usually have devastating consequences on habitats as well as on rare species through bycatches. For example, marine fishing operations often drastically reduce habitat complexity, alter productivity and remobilize contaminants and fine particulate matter [34, 35]. Therefore, the theoretical benefits of a selective harvesting program on animal *SR* are easily offset by the destruction of habitats and the precariousness of vulnerable populations. Nevertheless, the existence of the dominance-diversity trade-off highlights that gaining or losing individuals in a community will not have the same effects if these changes affect a few species only or the entire community.

The shared mathematical structure of RAD models could be worded as follows: “It is common to be rare”. The generality of this maxim in ecological communities, but also potentially in economic and social systems, has profound implications for the management of ecosystems. It underlines the presence of pervasive inequalities among resource users, which are only reshuffled by changing the environmental context, and thus, the rules of the apportionment game. It reminds us that strategies aiming solely at exploiting the natural capital, or at conserving species, are doomed to fail. Instead, protection of biodiversity could be promoted by encouraging a broad range of resource exploitation and socio-economical systems, each with its own set of rules.

## Supporting information

S1 Appendix Comparing models with and without replacing *Nmin* by 1 in Eq 2. (DOCX)
