# Supplementary material for: The dominance–diversity dilemma in animal conservation biology
Source: PLoS One. 2023 Mar 27;18(3):e0283439. doi: 10.1371/journal.pone.0283439 (PMC10042335; doi:10.1371/journal.pone.0283439)

**Appendix S1 - Comparing models with and without replacing *Nmin* by 1 in Eq. 2**

| Result | Replacing *Nmin* with 1 | Using actual *Nmin* value |
| --- | --- | --- |
| Pseudo-r^2^ | 0.68 | **0.73** |
| *MAE* | **1.66** | 1.69 |
| *MBE* | **1.56** | 1.64 |
| *cf* | **1.20** | 1.23 |
| *MAE** | 1.45 | **1.42** |
| *MBE** | **1.04** | 1.05 |

Fig. 1 replacing *Nmin* with 1


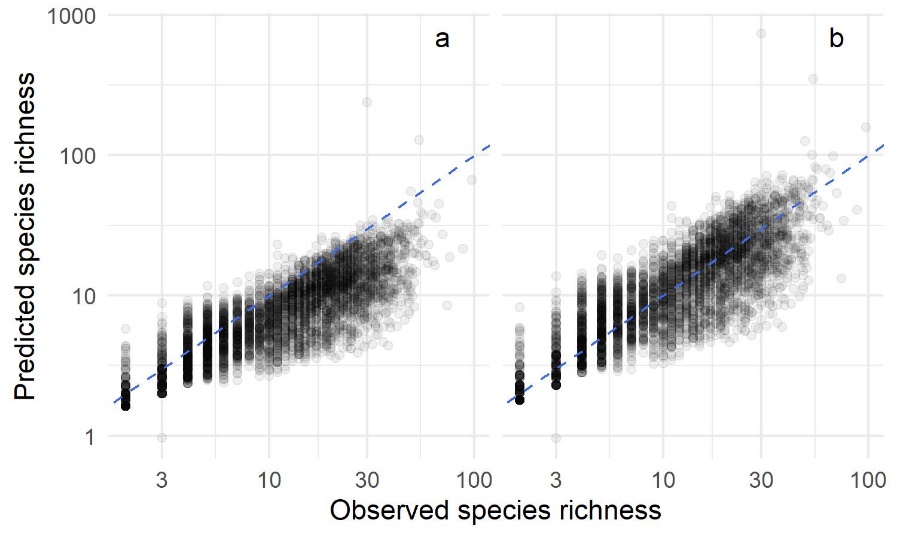


Fig. 1 using actual *Nmin* value.


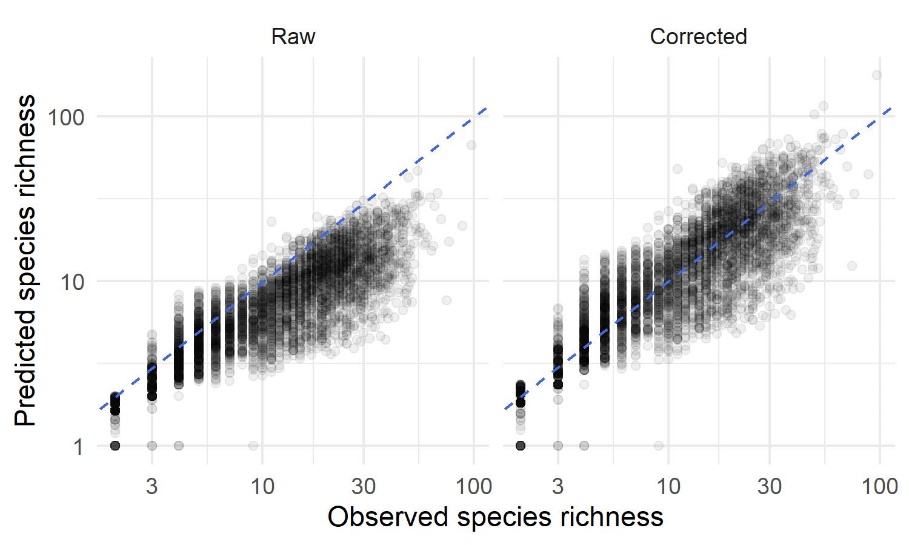

Supplement: S1 Appendix — (DOCX) [file pone.0283439.s001.docx]
